# Astaxanthin Mitigates 5-Fluorouracil-Induced Hepatotoxicity and Oxidative Stress in Male Rats

**DOI:** 10.3390/nu17071230

**Published:** 2025-04-01

**Authors:** Yasin Öztürk, Merve Öztürk, Muhammet Bahaeddin Dörtbudak, Francesca Mariotti, Gian Enrico Magi, Alessandro Di Cerbo

**Affiliations:** 1 Department of Pharmacology and Toxicology, Faculty of Veterinary Medicine, University of Necmettin Erbakan, Ereğli 42310, Konya, Türkiye; yasinozturk@erbakan.edu.tr; 2 Department Internal Medicine, Faculty of Veterinary Medicine, University of Necmettin Erbakan, Ereğli 42310, Konya, Türkiye; merve.ozturk@erbakan.edu.tr; 3 Department of Pathology, Faculty of Veterinary Medicine, Harran University, Şanlıurfa 63200, Şanlıurfa, Türkiye; mbdortbudak@gmail.com; 4 School of Biosciences and Veterinary Medicine, University of Camerino, 62024 Matelica, Italy; francesca.mariotti@unicam.it (F.M.); alessandro.dicerbo@unicam.it (A.D.C.)

**Keywords:** astaxanthin, 5-FU, hepatotoxicity, oxidative stress, biochemical parameters

## Abstract

Background: Hepatotoxicity, a significant complication of 5-fluorouracil (5-FU) treatment, is generally triggered by oxidative stress, liver damage, and apoptosis processes that take place in cancer patients. Methods: In this study, the protective effect of different astaxanthin (ASX) dosages (16 and 32/mg/kg/bw) was determined in rats with 5-FU-induced liver damage. Results: 5-FU induced a significant increase in the histopathological lesions severity and immunohistochemical (TNF-α and 8-OHdG) expression scores in the liver (*p* < 0.001), significantly increased serum liver parameters (AST, ALP, ALT, GGT, and TP) and malondialdehyde (*p* < 0.001), and, at the same time, significantly decreased antioxidant parameters (SOD, CAT, GST, GSR, Caspase-3, and GPx) (*p* < 0.001). Histopathological lesions and oxidative stress parameters significantly decreased in parallel while increasing the ASX dosage (*p* < 0.001). Conclusions: Based on these data, our results suggest that ASX may be considered a promising and valuable agent to mitigate hepatotoxicity and resistance mechanisms during cancer treatment.

## 1. Introduction

The prevalence of cancer is rising globally, and chemotherapeutic agents are employed as monotherapy or in combination with other agents in the management of cancer [1,2]. Due to the low therapeutic index of chemotherapeutics, they exert effects not only on malignant cells but also on healthy cells [3]. 5-fluorouracil (5-FU) is a representative of the antimetabolite chemotherapeutic family of drugs and is commonly utilized as a therapeutic agent against a range of cancers, including those of the colorectum, head and neck, breast, esophagus, pancreas, cervix, stomach, kidneys, and bladder, for which it has been in clinical use for decades [4]. Nevertheless, the utilization of this compound has been associated with significant toxicity and undesirable side effects, which have led to its classification as a hepatotoxic compound. Furthermore, it has been demonstrated to have hepatotoxic effects, with elevated levels of aspartate aminotransferase (AST), alkaline phosphatase (ALP), and evidence of liver involvement [4,5].

An increasing body of evidence has demonstrated that the tissue toxicity induced by 5-FU is associated with oxidative stress (OS) and inflammation caused by an increase in reactive oxygen species (ROS) [5]. Redox balance is a vital feature of the organism; disruption to this equilibrium, otherwise known as an imbalance of the redox system, is a defining characteristic of the disease process [1]. ROS are produced within the organism due to redox reactions from endogenous and exogenous sources. While ROS within physiological limits are essential for cell signaling, ROS within the pathological margin cause damage to cellular macromolecules, including lipids, nucleic acids, and proteins [6]. Enzymatic molecules, including superoxide dismutase (SOD), catalase (CAT), and glutathione peroxidase (GPx), are employed to neutralize excessive ROS [7]. The inability of antioxidant systems to maintain equilibrium during an increase in ROS results in OS [3]. The interplay between OS and inflammation results in the activation of apoptosis, ultimately leading to the irreversible damage and demise of tissue [1]. Given the absence of an approved treatment protocol or a specific antidote to be employed in the event of toxic effects resulting from the use of chemotherapy, several strategies to prevent toxicity are currently being investigated [6,8,9].

Astaxanthin (ASX), 3,30-dihydroxy-β,β-carotene-4,40-dione, is a naturally occurring xanthophyll carotenoid pigment with a red-orange hue that has been approved as a dietary supplement by the US Food and Drug Administration [10]. ASX has been demonstrated to possess potent antioxidant, anti-inflammatory, anti-cancer, and anti-apoptotic activities and the capacity to regulate gene expression [7,8,11,12]. Although ASX has been shown to diminish the toxicity of a range of chemotherapeutic agents in liver, brain, and kidney tissues [6,13,14], there is a paucity of research examining its impact on 5-FU-induced hepatotoxicity. This study aimed to investigate the therapeutic effect of ASX, known to possess antioxidant and anti-inflammatory properties, in a 5-FU-induced hepatotoxicity model. Additionally, this study represents the first investigation into the underlying mechanisms associated with this effect.

## 2. Materials and Methods

### 2.1. Experimental Animals and Study Design

The experiment protocol was designed in accordance with the guidelines and started after the Bingol University Animal Experiments Local Ethics Commission granted permission (Approval number: 25.01.2023-E.93970). Thirty male Wistar albino rats, 10–12 weeks old and weighing 250–300 g, were used in this experiment and fed ad libitum. Rats were kept in individual cages at room temperature of 25 ± 3 °C with 12 h dark/light cycles.

They had free access to standard laboratory feed (pellet food crushed to coarse powder) and water and were acclimatized for one week by maintaining hygienic conditions before beginning the experiment. To study the hepatoprotective effects of astaxanthin, rats were equally and randomly divided (https://www.randomizer.org/ (accessed on 20 February 2023)) into five groups (*n* = 6/group) and received different treatments:

Group I (control): 1 mL saline daily by gavage for 14 days;

Group II (5-FU): 5-FU (Kocak Pharma, Turkey) 100 mg/kg intraperitoneally on the first day;

Group III (ASX): astaxanthin 32 mg/kg/BW/day orally (dissolved in olive oil) every day for 14 days;

Group IV (ASX16): 5-FU 100 mg/kg/BW intraperitoneally on the first day + astaxanthin 16 mg/kg/BW/day orally (dissolved in olive oil) for 14 days;

Group V (ASX32): 5-FU at 100 mg/kg/BW intraperitoneally on the first day + astaxanthin 32 mg/kg/BW/day orally (dissolved in olive oil) for 14 days.

Animals were checked for weight gain, and food and water intake were measured daily. After 2 weeks, all animals were anesthetized using ketamine and xylazine and sacrificed, and the blood and livers were collected. Immediately after collection of the livers, they are stored at −20 °C for further studies. Blood was centrifuged at 5000 rpm for 10 min at 4 °C; then, the serum was transferred into microcentrifuge tubes (Eppendorf, Tarsons Products Pvt., Ltd., Kolkata, India) and stored at −20 °C until analyzed.

### 2.2. Liver Function Biomarkers Analysis

Liver function biomarkers such as aspartate aminotransferase (AST), alanine aminotransferase (ALT), alkaline phosphatase (ALP), gamma-glutamyl transferase (GGT), and total proteins (TP) were estimated in serum using a biochemical analyzer (Olympus AU-660, Tokyo, Japan) according to the manufacturer’s protocol.

### 2.3. Liver Pro-Oxidant/Antioxidant Biomarkers Analysis

The hepatic tissues were homogenized using TissueLyzer II^®^ (Qiagen, Germantown, MD, USA). The homogenates were centrifuged at 10,000× *g* for 20 min at 4 °C, and the obtained supernatants were analyzed. Caspase-3 (CASP-3) (Lot. No: 202212, Ref. DZE201110281) levels were assessed using the supernatants of liver homogenates, while superoxide dismutase (SOD) (Lot. No: 202209013), malondialdehyde (MDA) (Lot. No: 202212, Ref. DZE201110157), glutathione reductase (GSR) (Lot. No: 202212, Ref. DZE201115111), glutathione peroxidase (GPx) (Lot. No: 202212, Ref. DZE201111705), and glutathione-S-transferases (GST) (Lot. No: 202212, Ref. DZE201115110) levels in the serum were measured according to the manufacturer’s instructions using commercially available ELISA kits (Sunred Biological Technology Co., China). Samples were measured at 450 nm wavelength using a plate reader (Thermo Fisher Scientific, Waltham, MA, USA).

### 2.4. Histopathological Analysis

Liver tissue samples taken from all rats after necropsy were fixed in 10% neutral buffered formalin. The tissues were then routinely processed with increased concentrations of alcohols to paraffin blocks. Next, 4 µm thick tissue sections, obtained using a microtome (LEICA RM 2135, Wetzlar, Germany), were rehydrated and stained with hematoxylin and eosin to be examined under the light microscope (Olympus BX53, Japan). Two investigators blindly evaluated six different microscopic 20× magnification fields for each sample [2]. An overall histological score was attributed to each sample using semi-quantitative criteria, according to Canelli et al. 2023 with modifications [15] including the following histological parameters: (a) degenerative changes in the hepatocytes (0–4); (b) hepatocellular necrosis (0–4); (c) inflammatory cell infiltration (0–4); (d) hyperemia and hemorrhages (Table 1).

### 2.5. Immunohistochemical Analysis

Samples were submitted to immunohistochemical analysis using the antibodies anti-TNF (Tumor necrosis factor)-α and anti-8-OHDG (8-Hydroxyguanosine). Four-micron-thick sections, mounted on polylysine-coated slides, were deparaffinized and hydrated in a graded series of alcohols. Subsequently, endogenous peroxidase activity was blocked with 3% hydrogen peroxide; sections were treated for antigen retrieval. Nonspecific binding was blocked by incubating the sections with a protein block. Then, sections were incubated for 16 h with the following primary antibodies: mouse monoclonal antibody anti-TNF-α (SC-52B83, Santa Cruz, USA) and mouse monoclonal antibody anti-8-OHDG (SC-66036, Santa Cruz, CA, USA).

A goat anti-mouse biotinylated antibody was used as a secondary antibody. Subsequently, a standard ABC-peroxidase kit (Vector Laboratories) was used to visualize antibody–antigen binding. Rabbit IgG-isotype control (Abcam, Cambridge, UK) was used instead of primary antibodies for negative control. The immunohistochemical reaction was developed in 3,3-diaminobenzidine solution DAB (Sigma, Tokyo, Japan). The slides were counterstained with hematoxylin, dehydrated through alcohol series, and cleared in xylene before mounting. Sections were examined under light microscopy (Olympus BX53, Japan). Two blinded investigators assessed the immunostaining reaction, and the evaluation of immunopositivity was conducted in a semi-quantitative manner [16]. Briefly, the positive cell percentage was based on a 5-point scoring system: 0 = no positive cell, 1 = 20%, 2 = between 21% and 50%, 3 = between 51% and 70%, and 4 = 71%. A count of immunopositive and -negative cells was carried out for each microscopic field and then transformed into a percentage. The intensity of immunostaining was classified according to a 4-point scoring system: 0 = no staining, 1 = low-intensity staining, 2 = moderate-intensity staining, and 3 = high-intensity staining. In those samples with heterogeneous intensity, the chosen score was the predominant one in the sample itself. The overall score assigned to each case is derived by multiplying cell positivity and intensity signal score, with a minimum score of 0 and a maximum score of 12. Two authors evaluated the equivocal cases to establish the score.

### 2.6. Statistical Analysis

All the ELISA experiments were carried out in triplicate. Data were analyzed using GraphPad Prism 9 software (GraphPad Software, Inc., La Jolla, CA, USA). Data for liver and pro-oxidant/antioxidant biomarkers are presented as the means ± standard deviation (SD), while those for histopathological and immunohistochemical scoring are presented as boxes and whiskers (min to max). Differences in liver function and oxidative stress biomarkers were assessed using a One-Way Analysis of Variance (ANOVA) followed by the Tukey multiple comparison test. Conversely, differences among different histological and immunohistochemical scores were analyzed using a Kruskal–Wallis’s test followed by Dunn’s multiple comparison test. A *p* < 0.05 was considered significant.

## 3. Results

### 3.1. Liver Function Biomarkers

**Figure 1:**
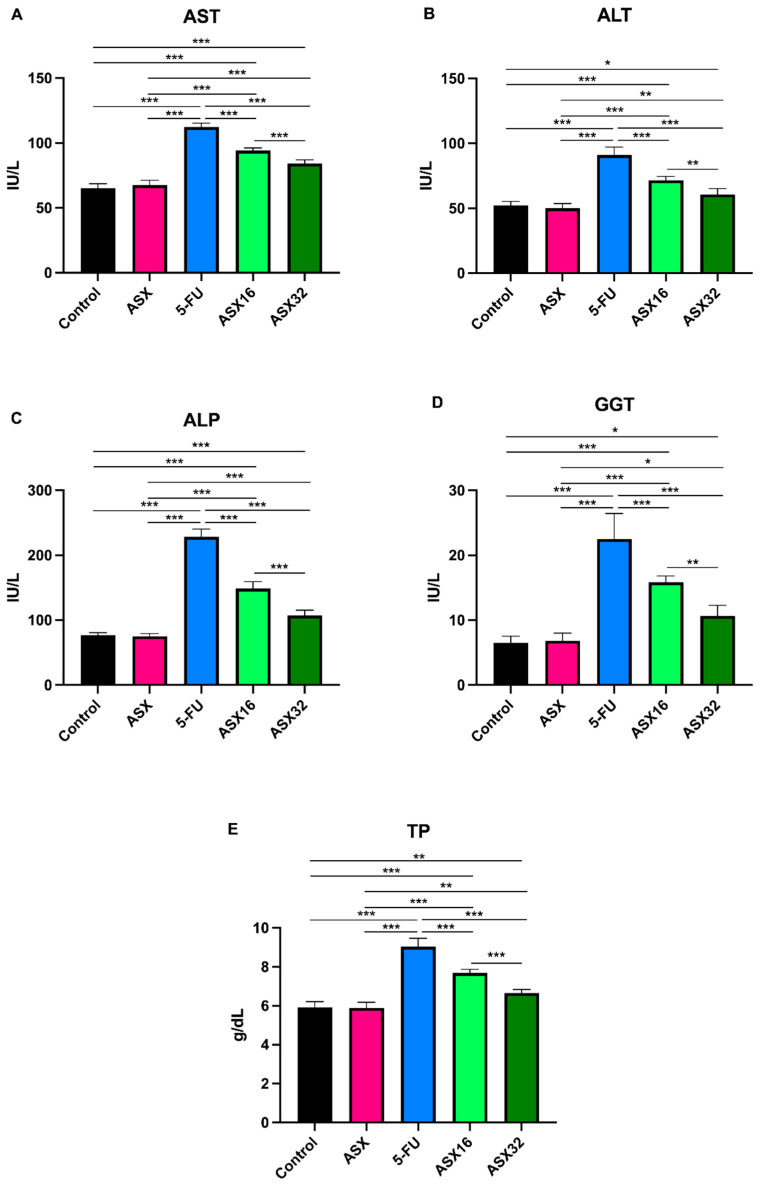
Graphical representation of serum liver function biomarkers’ trend following ASX (32 mg/kg), 5-FU, ASX16 (16 mg/kg + 5-FU), and ASX32 (32 mg/kg + 5-FU). *** *p* < 0.001, ** *p* < 0.01, and * *p* < 0.05. (**A**) aspartate aminotransferase (AST); (**B**) alanine aminotransferase (ALT); (**C**) alkaline phosphatase (ALP); (**D**) gamma-glutamyl transferase (GGT); (**E**) total proteins (TP).

Results concerning the liver function biomarkers’ analysis are summarized in Figure 1.

5-FU administration caused a significant increase (*p* < 0.001) in serum levels of all parameters compared to the control and ASX groups (from a mean value of 66.33 ± 3.68 IU/L to 112.3 ± 3.01 IU/L for AST (95% CI −52.48 to −41.85); from a mean value of 51.16 ± 3.17 IU/L to 91 ± 6.19 IU/L for ALT (95% CI −45.91 to −31.42); from a mean value of 75.92 ± 4.04 IU/L to 228.2 ± 11.92 IU/L for ALP (95% CI −166.0 to −137.0); from a mean value of 6.66 ± 1.1 IU/L to 22.5 ± 3.93 IU/L for GGT (95% CI −19.52 to −12.48); and from a mean value of 5.89 ± 0.29 g/dL to 9.03 ± 0.43 g/dL for TP (95% CI −3.612 to −2.621)).

At the same time, the ASX16 and ASX32 groups showed a significant mitigating effect with respect to the 5-FU group (*p* < 0.001) in a dose-dependent manner (from 112.3 ± 3.01 IU/L to 94.17 ± 2.04 IU/L (95% CI 12.85 to 23.48) and 84.17 ± 2.92 IU/L (95% CI 22.85 to 33.48), respectively, for AST; from 91 ± 6.19 IU/L to 71.50 ± 3.08 IU/L (95% CI 12.26 to 26.74) and 60.67 ± 4.54 IU/L (95% CI 23.09 to 37.58), respectively, for ALT; from 228.2 ± 11.92 IU/L to 148.5 ± 10.89 IU/L (95% CI 65.18 to 94.15) and 107 ± 8.34 IU/L (95% CI 106.7 to 135.7), respectively, for ALP; from 22.5 ± 3.93 IU/L to 15.83 ± 0.98 IU/L (95% CI 3.142 to 10.19) and 10.67 ± 1.63 IU/L (95% CI 8.309 to 15.36), respectively, for GGT; and from 9.03 ± 0.43 g/dL to 7.68 ± 0.18 g/dL (95% CI 0.8547 to 1.845) and 6.66 ± 0.18 g/dL (95% CI 1.888 to 2.879), respectively, for TP).

Nevertheless, significantly different increases were also observed in the ASX16 and ASX32 groups with respect to control and ASX groups for the various parameters. In particular, all parameters showed a similar significance trend (*p* < 0.001) when dealing with ASX16 (from 65.17 ± 3.54 IU/L of control and 65.70 ± 3.83 IU/L of ASX to 94.17 ± 2.04 IU/L of ASX16 for AST (95% CI −34.31 to −23.69 and −31.98 to −21.35, respectively); from 52.33 ± 2.94 IU/L of control and 50.0 ± 3.74 IU/L of ASX to 71.5 ± 3.08 IU/L for ALT (95% CI −26.41 to −11.92 and −28.74 to −33.76, respectively); from 76.67 ± 3.93 IU/L of control and 75.17 ± 4.16 IU/L of ASX to 148.5 ± 10.89 IU/L for ALP (95% CI −86.32 to −57.35 and −87.82 to −58.85, respectively); from 6.50 ± 1.05 IU/L of control and 6.83 ± 1.17 IU/L of ASX to 15.83 ± 0.98 IU/L for GGT (95% CI −12.86 to −5.809 and −12.52 to −5.476, respectively); from 5.91 ± 0.30 g/dL of control and 5.88 ± 0.29 g/dL of ASX to 7.68 ± 0.18 g/dL for TP (95% CI −2.262 to −1.271 and −2.295 to −1.305, respectively)).

When dealing with the ASX32 group, the significance was different among parameters, even compared to the control or the ASX group. For instance, AST and ALP showed a similar significant increase (*p* < 0.001) in the ASX32 group if compared to the control or the ASX group (from 65.17 ± 3.54 IU/L of control and 65.70 ± 3.83 IU/L of ASX to 84.17 ± 2.92 IU/L for AST (95% CI −24.31 to −13.69 and −21.98 to −11–35, respectively); from 76.67 ± 3.93 IU/L of control and 75.17 ± 4.16 IU/L to 107 ± 8.43 IU/L for ALP (95% CI −44.82 to −15.85 and −46.32 to −17.35, respectively). A less significant increase was observed in ALT (from 52.33 ± 2.94 IU/L of control and 50 ± 3.74 IU/L of ASX to 60.67 ± 4.54 IU/L, *p* < 0.05 and *p* < 0.01, respectively (95% CI −15.58 to −1.09 and −17.91 to −3.424, respectively)). As for GGT, a similar significant increase (*p* < 0.05) in the ASX32 group was observed (from 6.50 ± 1.05 IU/L of control and 6.83 ± 1.17 IU/L of ASX to 10.67 ± 1.63 IU/L (95% CI −7.691 to −06424 and −7.358 to −0.3091, respectively)). Also, TP showed a similar significant increase (*p* < 0.01) in the ASX32 group (from 5.91 ± 0.30 g/dL of control and 5.88 ± 0.29 g/dL of ASX to 6.65 ± 0.18 g/dL (95% CI −1.229 to −02380 and −1.262 to −0.2713)).

### 3.2. Liver Pro-Oxidant/Antioxidant Biomarkers

**Figure 2:**
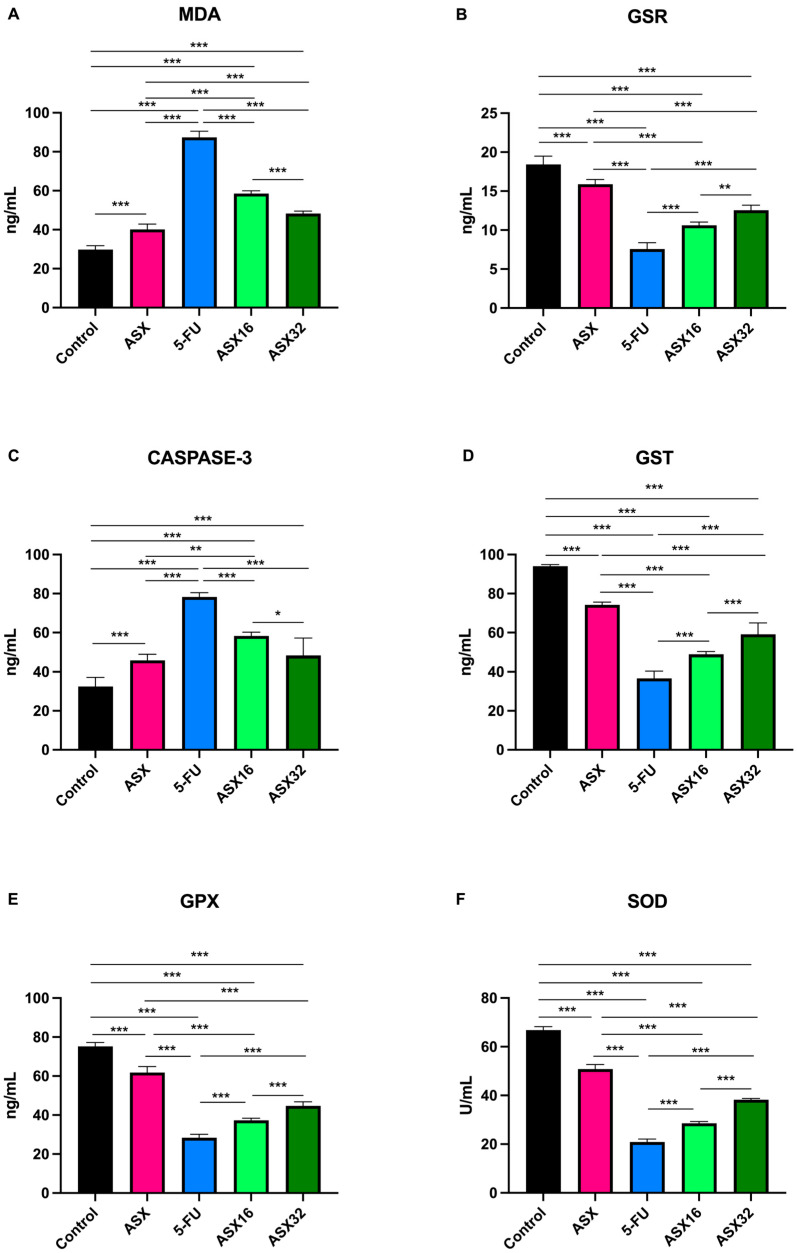
Graphical representation of serum oxidative stress biomarkers’ trend following ASX (32 mg/kg)**,** 5-FU, ASX16 (16 mg/kg + 5-FU), and ASX32 (32 mg/kg + 5-FU). *** *p* < 0.001 and ** *p* < 0.01, * *p* < 0.05. (**A**) malondialdehyde (MDA); (**B**) glutathione reductase (GSR); (**C**) Caspase-3 (CASP-3); (**D**) glutathione-S-transferases (GST); (**E**) glutathione peroxidase (GPx); (**F**) superoxide dismutase (SOD).

Results concerning the oxidative stress biomarkers’ analysis are summarized in Figure 2.

5-FU administration caused a significant increase (*p* < 0.001) in serum levels of MDA and CASPASE-3 (from 29.75 ± 2.08 ng/mL of control and 40.10 ± 2.81 ng/mL of ASX to 87.35 ± 3.18 ng/mL for MDA (95% CI −61.47 to −53.73 and −51.13 to −43.38, respectively), and from 32.42 ± 4.69 ng/mL of control and 45.87 ± 3.01 ng/mL of ASX to 78.32 ± 2.12 ng/mL (95% CI −54.15 to −37.65 and −40.70 to −24.20, respectively) for CASPASE-3) and a significant decrease (*p* < 0.001) in serum levels of the other parameters (from 18.42 ± 1.08 ng/mL of control and 15.89 ± 0.6 ng/mL of ASX to 7.56 ± 0.81 ng/mL of GSR (95% CI 9.583 to 12.12 and 7.057 to 9.593, respectively); from 94 ± 0.89 ng/mL of control and 74.17 ± 1.47 ng/mL of ASX to 36.60 ± 3.8 ng/mL of GST (95% CI 51.85 to 62.95 and 32.02 to 43.11, respectively); from 75.23 ± 1.99 ng/mL of control and 61.82 ± 3.05 ng/mL of ASX to 28.38 ± 1.73 ng/mL of GPx (95% CI 43.33 to 50.37 and 29.91 to 36.96) and from 66.80 ± 1.46 ng/mL of control and 50.80 ± 1.89 U/mL of ASX to 20.90 ± 1.19 U/mL of SOD (95% CI 43.76 to 48.04 and 27.76 to 32.04, respectively)).

At the same time, the ASX16 and ASX32 groups showed a significant mitigating effect with respect to the 5-FU group (*p* < 0.001) in a dose-dependent manner (from 87.35 ± 3.18 ng/mL to 58.55 ± 1.42 ng/mL and 48.27 ± 1.28 ng/mL, respectively, for MDA (95% CI 24.93 to 32.67 and 35.21 to 42.95, respectively); from 7.56 ± 0.81 ng/mL to 10.62 ± 0.41 ng/mL and 12.55 ± 0.64 ng/mL, respectively, for GSR (95% CI −4.318 to −1.782 and −6.252 to −3.715, respectively); from 78.32 ± 2.12 ng/mL to 58.32 ± 1.9 ng/mL and 48.27 ± 8.9 ng/mL, respectively, for CASPASE-3 (95% CI 11.75 to 28.25 and 21.80 to 38.30, respectively); from 36.6 ± 3.8 ng/mL to 48.83 ± 1.47 ng/mL and 59.12 ± 5.82 ng/mL, respectively, for GST (95% CI −17.78 to −6.685 and −28.06 to −16.97, respectively); from 28.38 ± 1.73 ng/mL to 37.28 ± 1.05 ng/mL and 44.72 ± 2.03 ng/mL, respectively, for GPx (95% CI −12.42 to −5.377 and −19.86 to −12.81, respectively) and from 20.90 ± 1.19 U/mL to 28.58 ± 0.69 U/mL and 38.20 ± 0.55 U/mL, respectively, for SOD (CI −9.820 to −5.546 and −19.44 to −15.16, respectively)).

Nevertheless, a significant increase (*p* < 0.001) was observed in the ASX16 and ASX32 groups with respect to the control for MDA (from 29.75 ± 2.08 ng/mL to 58.55 ± 1.42 ng/mL and 48.27 ± 1.28 ng/mL, respectively, (95% CI −32.67 to −24.93 and −22.39 to −14.65, respectively)) and CASPASE-3 (from 32.42 ± 4.69 ng/mL to 58.32 ± 1.9 ng/mL and 48.27 ± 8.9 ng/mL, respectively (95% CI −34.15 to −17.65 and −24.10 to −7.595, respectively)). Dealing with the ASX group, a significantly different increase with respect ASX16 and ASX32 groups was observed for MDA (from 40.10 ± 2.81 ng/mL to 58.55 ± 1.42 ng/mL and 48.27 ± 1.28 ng/mL, respectively, *p* < 0.001 (95% CI −22.33 to −14.58 and −12.04 to −4.302, respectively)) and for CASPASE-3 (from 45.87 ± 3.01 ng/mL to 58.32 ± 1.9 ng/mL for ASX16, *p* < 0.01 (95% CI −20.70 to −4.195)).

Conversely, the other parameters showed a similar trend (*p* < 0.001) with a significant decrease in the ASX16 and ASX32 groups with respect to the control (from 18.42 ± 1.08 ng/mL and 15.89 ± 0.6 ng/mL to 10.62 ± 0.41 g/dL (95% CI 6.533 to 9.070 and 4.600 to 7.137, respectively) for GSR; from 94 ± 0.89 ng/mL and 74.17 ± 1.47 ng/mL to 48.83 ± 1.47 ng/mL (95% CI 39.62 to 50.71 and 29.34 and 40.43, respectively) for GST; from 75.23 ± 1.99 ng/mL and 61.82 ± 3.05 ng/mL to 37.28 ± 1.05 ng/mL (95% CI 34.43 to 41.47 and 26.99 to 34.04, respectively) for GPx and from 66.80 ± 1.46 U/mL and 50.80 ± 1.89 g/dL to 28.58 ± 0.69 U/mL (95% CI 36.08 to 40.35 and 26.46 to 30.74, respectively) for SOD) and ASX (from 18.42 ± 1.08 ng/mL and 15.89 ± 0.6 ng/mL to 12.55 ± 0.64 ng/mL (95% CI 4.007 to 6.543 and 2.073 to 4.610, respectively) for GSR; from 94 ± 0.89 ng/mL and 74.17 ± 1.47 ng/mL to 59.55 ± 0.64 ng/mL (95% CI 19.79 to 30.88 and 9.502 to 20.60, respectively) for GST; from 75.23 ± 1.99 ng/mL and 61.82 ± 3.05 ng/mL to 44.72 ± 2.03 ng/mL (95% CI 21.01 to 28.06 and 12.58 to 20.62, respectively) for GPx and from 66.80 ± 1.46 U/mL and 50.80 ± 1.89 U/mL to 38.20 ± 0.55 U/mL (95% CI 20.08 to 24.35 and 10.46 to 14.74, respectively) for SOD).

### 3.3. Histopathological Analysis

**Figure 3:**
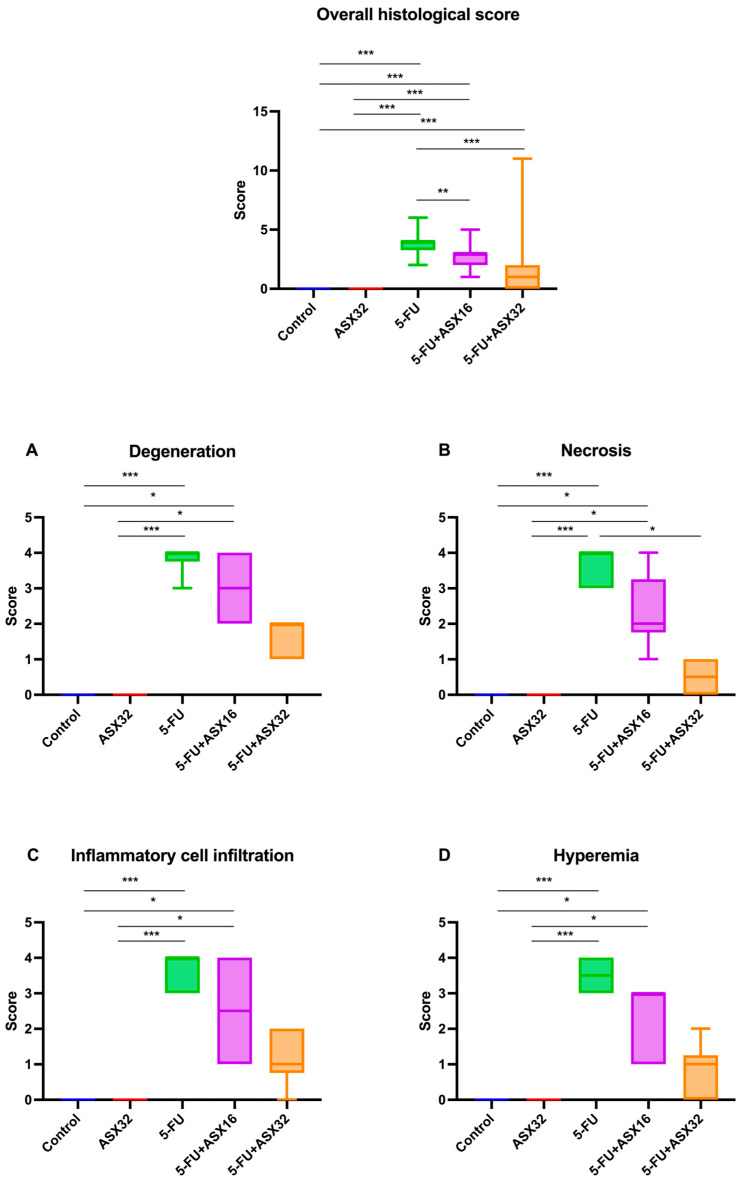
Graphical representation of the histopathological lesion’s severity score in experimental groups (*n* = 6/each group). *** *p* < 0.001 and ** *p* < 0.01, * *p* < 0.05. (**A**) degeneration, (**B**) necrosis, (**C**) inflammatory cell infiltration, and (**D**) hyperemia.

The results of the histological score comparing different groups are shown in Figure 3.

Considering the overall histological score, statistical differences in terms of the severity of lesions were observed between the control and 5-FU groups (*p* < 0.001), control and ASX16 + 5-FU groups (*p* < 0.001), control and ASX32 + 5-FU groups (*p* < 0.001), ASX32 and 5-FU groups (*p* < 0.001), ASX32 and ASX16 + 5-FU groups (*p* < 0.001), 5FU and ASX16 + 5-FU groups (*p* < 0.001), and 5-FU and ASX32 + 5-FU groups (*p* < 0.001).

Regarding specific histological parameters, such as presence of inflammatory cell hepatocyte degeneration and necrosis, inflammatory cell infiltration, and hyperemia, a statistical difference in the score between the control and 5-FU groups (*p* < 0.001), control and ASX16 + 5-FU groups (*p* < 0.05), ASX32 and ASX16 + 5-FU groups (*p* < 0.05), and ASX32 and 5-FU groups (*p* < 0.001) was observed. For hepatocyte necrosis, there was also a statistical difference in the score between the 5-FU and ASX32 + 5-FU groups (*p* < 0.05).

**Figure 4:**
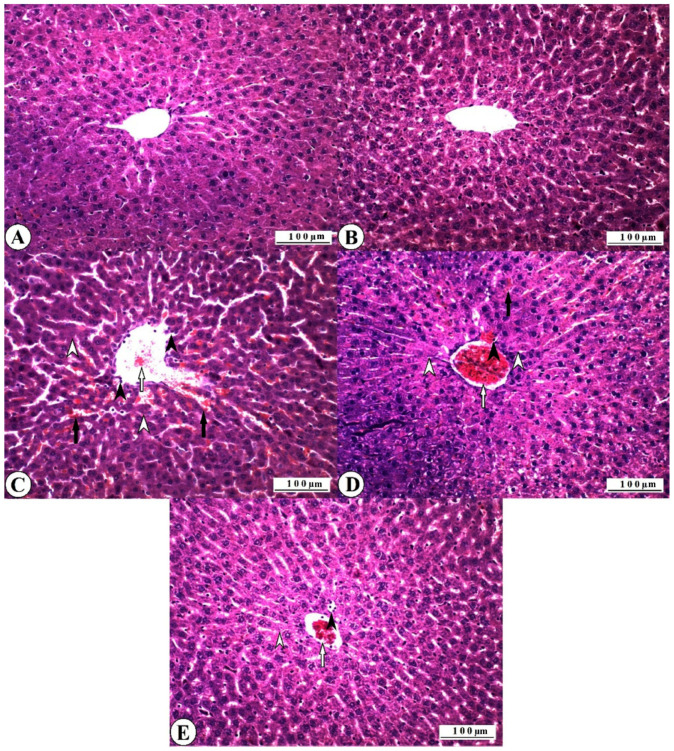
Histopathological findings in experimental groups: liver, HE, X200. Bar: 100 µ. (**A**) Control group, normal histological appearance. (**B**) ASX32 group, normal histological appearance. (**C**) 5-FU group, degenerative and necrotic changes (hollow arrowheads), inflammatory cell infiltration (arrowheads), vascular hyperemia (hollow arrow), and sinusoidal hemorrhage (arrows). (**D**) ASX16 + 5-FU group, degenerative and necrotic changes (hollow arrowheads), inflammatory cell infiltration (arrowhead), vascular hyperemia (hollow arrow), and sinusoidal hemorrhage (arrow). (**E**) ASX32 + 5-FU group, degenerative and necrotic changes (hollow arrowhead), inflammatory cell infiltration (arrowhead), and vascular hyperemia (hollow arrow).

Histopathological findings observed in all experimental groups are reported in Figure 4.

Regular histomorphological features were recorded in all control group animals, and no histopathological lesions were observed (Figure 4A). Normal histology was also observed in all animals in the ASX group (Figure 4B). Severe pathological changes were observed in the liver tissues of all 5-FU group animals. Severe degenerative changes were recorded in the liver hepatocytes, especially in the perivascular cells. These were mainly in the form of vacuolar degeneration, and partially fatty changes were observed.

Coagulative necrosis was observed, especially in the periacinar zone, where the degeneration was severe. Dissociation was observed in the radial alignment of the hepatocytes due to degenerative and necrotic changes. Vascular changes accompanied the destruction of the tissue elements, and hyperemia and partial hemorrhages were observed in the vessels. Numerous polymorphonuclear cellular infiltrations were observed in the vascular lumens and sinusoidal spaces (Figure 4C). Histopathological lesions were observed in all animals of the ASX16 + 5-FU group. However, the severity of these lesions was found to be less than the findings of the 5-FU group.

Severe degeneration was observed in hepatocytes, and necrotic changes were less severe than in the 5-FU group. Although hyperemia was severe in the vessels, hemorrhages were not observed. Inflammatory cell infiltration was less than in the 5-FU group (Figure 4D). It was determined that pathological changes were relatively less in the ASX32 + 5-FU group than in the 5-FU group and partially better than in the ASX16 + 5-FU group. Although necrotic lesions were not observed, degenerative changes were commonly observed in the hepatocytes. While hemorrhagic foci were not observed, mild hyperemia was observed. Inflammatory cell infiltration was lower than in the ASX16 + 5-FU, especially in the 5-FU group (Figure 4E).

### 3.4. Immunohistochemical Analysis

**Figure 5:**
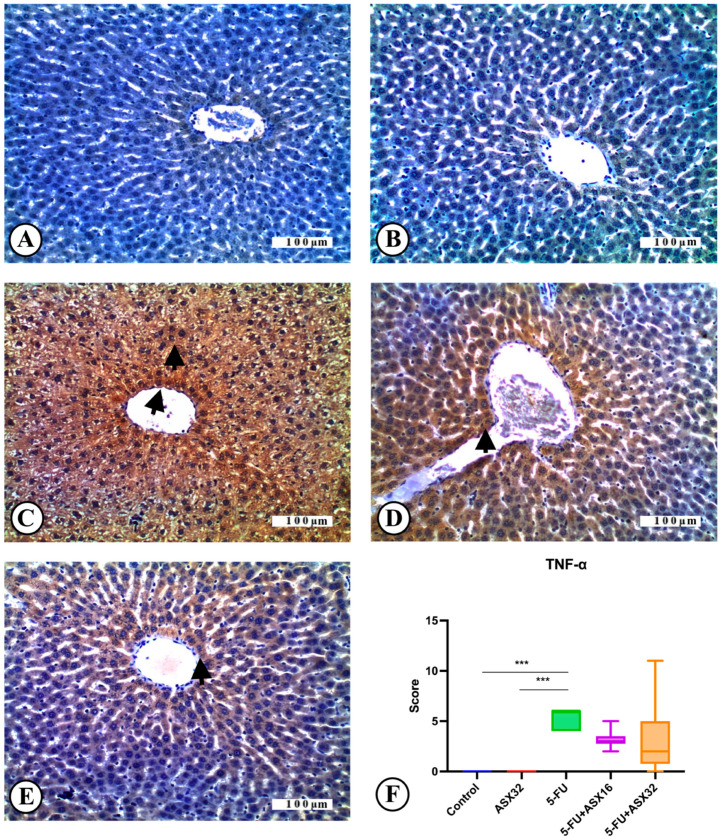
TNF-α immunoreactions in experimental groups: liver, IHC, X200. Bar: 100 µ. *** *p* < 0.001. (**A**) Control group, TNF-α immunonegative. (**B**) AST32 group, TNF-α immunonegative. (**C**) 5-FU group, severe TNF-α expression (arrowheads). (**D**) AST16 + 5-FU group, moderate TNF-α expression (arrowhead). (**E**) AST32 + 5-FU group, mild TNF-α expression (arrowhead). (**F**) TNF-α expression score.

Immunohistochemical findings for TNF-α expression in all experimental groups are reported in Figure 5.

TNF-α expression was not observed in the control group (*n* = 6) (Figure 5A). Similarly, no immune positivity was recorded regarding TNF-α in the ASX group (*n* = 6) (Figure 5B). Severe TNF-α expression was observed in the liver tissue sections of the 5-FU group (*n* = 6) (Figure 5C). It was observed that TNF-α expression decreased in the ASX16 + 5FU group (*n* = 6) compared to the 5-FU group (Figure 5D). Moderate TNF-α expression was observed in the ASX32 + 5FU group (*n* = 6), although it was less compared to the ASX16 and the 5-FU groups (Figure 5E). In line with these findings, it was observed that TNF-α expression, one of the important and frequently preferred biomarkers of inflammation, was severe in the 5-FU group, and TNF-α expression decreased in parallel with the increase in the dose of ASX application. Regarding the immunohistochemical TNF-α expression score, there was a statistical difference between the control and 5-FU groups and between the ASX32 and 5-FU groups (Figure 5F).

**Figure 6:**
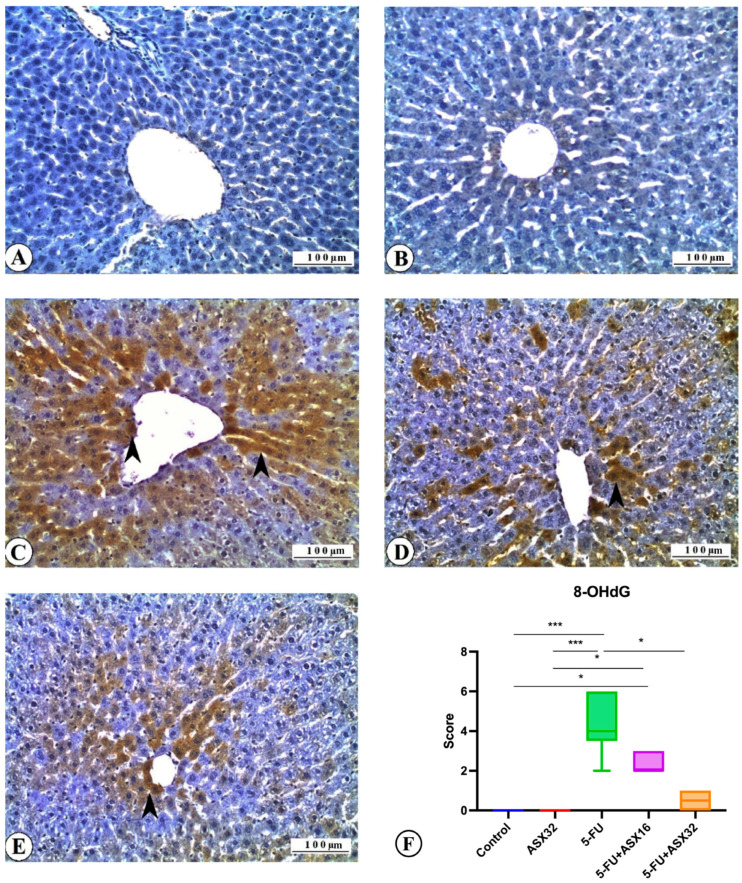
8-OHdG immunoreactions in experimental groups: liver, IHC, X200. Bar: 100 µ. *** *p* < 0.001, * *p* < 0.05. (**A**) Control group, 8-OHdG immunonegative. (**B**) ASX group, 8-OHdG immunonegative. (**C**) 5-FU group, severe 8-OHdG expression (arrowheads). (**D**) ASX16 group, moderate 8-OHdG expression (arrowhead). (**E**) ASX32 group, mild 8-OHdG expression (arrowhead). (**F**) 8-OHdG expression score.

Immunohistochemical findings for 8-OHdG expression in all experimental groups are reported in Figure 6.

No 8-OHdG expression was observed in the control group (Figure 6A). Similarly, no immunostaining was recorded in the ASX group for 8-OHdG (Figure 6B). Severe 8-OHdG expression was observed in liver tissue sections of the 5-FU group (Figure 6C). It was also observed that 8-OHdG expression decreased in the ASX16 + 5FU and ASX32 + 5FU groups compared to the 5-FU group (Figure 6D,E). In line with these findings, it was seen that 8-OHdG expression, one of the important biomarkers used in determining DNA oxidation, i.e., DNA damage, was marked in the 5-FU group and decreased along with the increase in ASX dose. It was also determined that ASX alone did not cause 8-OHdG expression. The DNA damage expression score of 8-OHdG is shown in Figure 6F. Statistical differences between the control and 5-FU groups (*p* < 0.001), control and ASX16 + 5-FU groups (*p* < 0.05), ASX32 and 5-FU groups (*p* < 0.001), ASX32 and ASX16 + 5-FU groups (*p* < 0.05), and 5-FU and ASX32 + 5-FU groups (*p* < 0.05) were observed.

## 4. Discussion

The liver is the central organ involved in the detoxification of metabolic waste. Hepatotoxicity is the most important side effect of many chemotherapeutic agents used to treat cancer [2]. 5-FU, one of the most important drugs used in the treatment of cancer, is extensively metabolized in the liver, and the toxic metabolites produced lead to severe hepatotoxicity, which limits the chemotherapeutic effect of 5-FU. 5-FU-induced hepatotoxicity causes inflammation, oxidation, and apoptosis [17,18,19]. This study aimed to evaluate the effects of two different dosages of ASX on hematochemical (AST, ALT, ALP, GGT, and TP), oxidative (SOD, MDA, GSR GPx, GST, and CASP-3), and anti-inflammatory (TNF-α and 8-OHDG) markers involved in the pathological processes of 5-FU-induced hepatotoxicity and on histological liver status [20].

The administration of 5-FU resulted in a significant increase in the levels of all aforementioned parameters compared to the control group, possibly due to an overproduction of inflammatory mediators and ROS, contributing to liver damage and apoptosis phenomenon onset [21,22].

However, the co-administration of ASX, in particular at 32 mg/kg, significantly mitigated such toxic effects. Nevertheless, we could not observe a complete restoration of the parameter level; we therefore hypothesized that this phenomenon could be ascribed to the short half-life of ASX (367 min) and to its rapid decline to < 0.02 μg/mL observed after 8 h following intragastric administration at a dose of 20 mg/kg [23].

Despite this last limitation, the results proved the hepatoprotective activity of ASX by reducing hepatic damage and dysfunction [2,24,25], possibly through an interplay between the nuclear factor erythroid 2-related factor 2 (Nrf2) and the nuclear factor kappa-light-chain enhancer of activated B cells (NF-κB) signaling networks [11,26,27]. In this sense, ASX has been shown to suppress proinflammatory IL-6 expression through p-ERK1/2-MSK-1- and p-NF-κB p65-dependent pathways [28], preventing oxidative damage through the activation of the phosphoinositide 3-kinase (PI3K/AKT) signaling pathway, which is involved in 8-OHDG reduction [29] and extracellular signal-regulated protein kinase (ERK) [11,24,26].

Despite the p-NF-κB p65-dependent pathway being known to repress the *Nrf2*-antioxidant response elements (AREs) at the transcriptional level [30], their activation, and consequent improvement in the antioxidant response, may be due to the facilitated Nrf2 dissociation from the Kelch-like ECH-associated protein 1 (Keap1) following the activation of PI3K/AKT and ERK by ASX [11,24,31]. In particular, many studies showed that the ERK pathway is involved in the activation of *Nrf2* in the cell cytoplasm, its dissociation from Keap1, and translocation into the nucleus to regulate AREs, such as HO-1, SOD, CAT, and GPX [28,32,33,34,35], whose expression is positively correlated to the of the antioxidant enzyme/DNA repair system and liver damage reversion [36,37,38,39].

As reported for doxorubicin, 5-FU could inactivate the ERK cascade, in particular ERK1/2, preventing Nrf2 nuclear translocation and the consequent antioxidant response, while ASX could revert this mechanism by significantly increasing the level of ERK1/2 [35,40,41,42].

The improvement in the antioxidant response, as indicated by the trend of SOD, MDA, GSR GPx, and GST, was significantly higher for ASX32 than that observed for ASX16, thus confirming the need for high dosages observed by other authors to achieve a regression trend towards control values [9]. In vitro studies have shown that ASX is a free radical scavenger with a potency several times greater than that of β-carotene and α-tocopherol [14,43,44], conferring a protective role against cancer [45] and ulcers [46]. Moreover, it has been shown that electron transfer from isoflavonoids formed during oxidative stress to carotenoid radical cation is faster in ASX than in other carotenoids [47], helping the lipid membranes in resisting against chain reactions of fatty acids oxidation [48]. Recently, ASX demonstrated a role in the ferroptosis, a novel form of programmed cell death [49,50] strongly associated with drug-induced liver injury and driven by iron-dependent lipid peroxidation [51,52,53]. In fact, ferroptosis involves a vicious circle characterized by intracellular iron overload followed by ROS production increase, redox balance impairment, and finally by oxidative stress and inflammatory response that led to increased tissue damage and further ferroptosis promotion [53]. In this sense, ASX has been proposed to relieve ferroptosis by activating the P53/SLC7A11/GPX4 pathway, downregulating P53, increasing *SLC7A1* and GPX4 gene transcription, and reducing oxidative stress via ROS scavenging activity [54,55].

Besides its antioxidant activity, ASX32 significantly mitigated the increased level of CASP-3, a crucial biomarker in the apoptosis mechanism, following the 5-FU challenge, underscoring the severity of liver tissue damage and confirming its protective effect on DNA damage [3,14].

Additionally, the histopathologic and immunohistochemical findings of the liver, supported by statistical analysis, revealed a beneficial dose-dependent effect of ASX in the groups intraperitoneally injected with 5-FU. The observed pathological changes were less severe than in the 5-FU group. These findings are in accordance with a study carried out to investigate the hepatoprotective role of astaxanthin from aflatoxin B1-induced toxicity in rats [56]. Degenerative and necrotic changes of the hepatocytes were reduced in the group treated with ASX, as well as DNA oxidation determined by 8-0HdG immunohistochemical expression, suggesting a protective role of ASX from the degenerative process through scavenging activity against peroxyl radicals.

Moreover, despite the protective role of ASX against hepatic inflammation, apoptosis, and oxidative stress, it is reasonable to hypothesize a protective role against the resistance mechanism that generally occurs during long-term treatment with 5-FU [1,57,58,59]. The main long-term implication of repeated 5-FU use is the resistance mechanism regulated by folypolyglutamate [57,58] and thymidylate synthase (*TYMS*) [1,59]. In the first case, the resistance mechanism is related to a decreased expression of folypolyglutamate synthetase, which is involved in the homeostasis and the survival of proliferating cancer cells. As for *TYMS,* it is considered the primary site of action for 5-fluorodeoxyuridine monophosphate (5-FdUMP), one of the three metabolites of 5-FU and, along with 5,10-methylene tetrahydrofolate, forms a ternary complex that blocks the access of dUMP to the nucleotide-binding site of *TYMS* by competition with FdUMP, resulting in a deoxynucleotides imbalance, such as deoxyuridine triphosphate (dUTP), that leads to DNA damage in cancer cells [1,60]. TYMS is also upregulated by the forkhead box M1 transcription factor, which is overexpressed during the onset of the resistance mechanism. The ASX could play a pivotal role in preventing such a mechanism due to its demonstrated activity on Nrf2, a TYMS inhibitor [32,61]. At the same time, Kavitha et al. (2013) demonstrated the ability of ASX to prevent the resistance mechanism by attenuating NF-κB signaling through a suppression of IKKβ and, subsequently, the restraint of the phosphorylation and degradation of IκB-α, thus blocking NF-κBp65 nuclear translocation [62]. Besides NF-κB signaling attenuation, ASX was associated with the inhibition of GSK-3β, a key component of Wnt pathway that, through an Akt-mediated phosphorylation, promotes β-catenin nuclear translocation from cytosol and transactivation of genes involved in cell proliferation and apoptosis evasion (Bcl-2, p-Bad, and survivin) [63]. Similar to our study, Kavitha et al. (2013) also correlated ASX to caspase-mediated mitochondrial apoptosis by the Akt-mediated dephosphorylation of proapoptotic Bax and Bad, which bind to and inhibit the Bcl-2 family of antiapoptotic proteins; enforced nuclear localization of survivin, enabling the efflux of Smac/Diablo and cytochrome-c from the mitochondria into the cytosol; and poly (ADP-ribose) polymerase (PARP) cleavage [62].

Last but not least, our results also offer a great opportunity for patients with advanced cancer undergoing palliative cytotoxic chemotherapy, which in most cases negatively impacts their quality of life [64]. In this sense, the prophylactic use or co-administration of ASX32 in the form of tablets, capsules [65], or encapsulation within liposomes [66] could maximize the therapy’s benefit, minimize the related risks, improve the quality of life, reduce symptom burden, and prolong patients’ survival.

## 5. Conclusions

This study demonstrated that ASX could significantly mitigate/restore liver damage induced by 5-FU in rats. It can be postulated that ASX may possess therapeutic potential against 5-FU-induced liver hepatotoxicity during cancer treatment, preventing the onset of 5-FU resistance mechanisms and improving the patients’ quality of life. Nevertheless, further in vivo studies are required to substantiate the safe use of ASX over a long period.

## Figures and Tables

**Table 1:** Histological scoring parameters for the evaluation of 5 FU hepatotoxicity.

Histological Feature	Score	Description
Degenerative changes in the hepatocytes	0	none
	1	minimal
	2	mild
	3	moderate
	4	severe
Hepatocellular necrosis in the periacinar zone	0	none
	1	minimal
	2	mild
	3	moderate
	4	severe
Inflammatory cell infiltration	0	none
	1	minimal
	2	mild
	3	moderate
	4	severe
Hyperemia and hemorrhages	0	none
	1	minimal
	2	mild
	3	moderate
	4	severe

## Data Availability

The data presented in this study are available on request from the corresponding author due to privacy reasons.

## Abbreviations

The following abbreviations are used in this manuscript:

ROS	Reactive oxygen species
5-FU	5-fluorouracil
AST	Aminotransferase
ALP	Alkaline phosphatase
SOD	Superoxide dismutase
CAT	Catalase
GPx	Glutathione peroxidase
MDA	Malondialdehyde
GSR	Glutathione reductase
GGT	Gamma-glutamyl transferase
TP	Total protein
TNF-α	Tumor necrosis factor-α
ASX	Astaxanthin
8-OHDG	8-Hydroxyguanosine
